# Ecmo-assisted carinal resection and reconstruction after left pneumonectomy

**DOI:** 10.1186/1749-8090-5-89

**Published:** 2010-10-20

**Authors:** Jie Lei, Kai Su, Xiao F Li, Yong A Zhou, Yong Han, Li J Huang, Xiao P Wang

**Affiliations:** 1 Department of Thoracic Surgery, Tang Du Hospital, Fourth Military Medical University, Xi'an, Shaanxi 710038, China

## Abstract

Extracorporeal Membrane Oxygenation (ECMO) has become an increasingly important technique for patients with respiratory or cardiac failure for a variety of causes. In addition, there are many reports about the use of ECMO in surgical operation on neonates and children patients with tracheal obstruction. In this report we present a case about an adult patient who underwent a carinal resection and reconstruction after left pneumonectomy with ECMO assistance successfully. To our knowledge, this case is the first of its kind to use ECMO in adult carinal resection and reconstruction after pneumonectomy. In this report, we try to illustrate that ECMO is effective in operations of this kind.

## Background

Extracorporeal membrane oxygenation (ECMO), a form of artificial circulatory support, is currently being used in ICUs worldwide to support patients with respiratory or cardiac failure who are unresponsive to conventional therapeutic interventions, but it hardly ever used as auxiliary implement to lung cancer surgery, especially in the secondary surgery. Herein we report a case attempt in this rare, new surgical procedure in the successful resection and reconstruction of carina after left pneumonectomy.

## A Case Presentation

**Figure 1:**
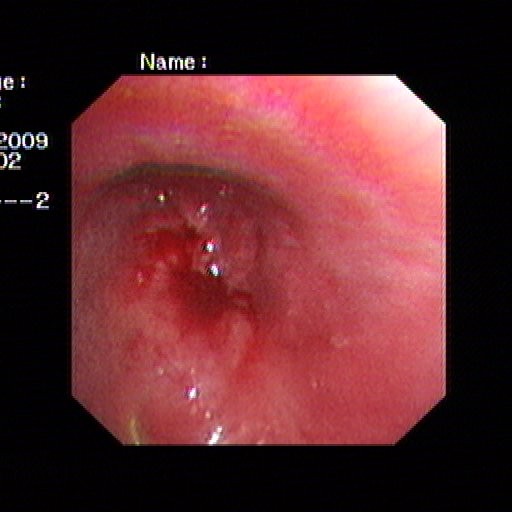
**Bronchoscopic findings showing that the left postpneumonectomy stump was filled with blood and the mucous membrane of it was rough**.

**Figure 2:**
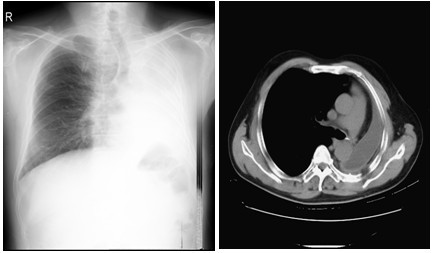
**Chest radiograph(Left) and Chest CT (Right) demonstrating the left lung was excised and no lymphatic metastasis**.

A 55-year-old man presented himself complaining of intermittent hemoptysis and short breath for two week. His medical history included squamous cell lung cancer of the left main stem bronchus involving the inferior lobe orifice, treated with left pneumonectomy and chemotherapy nearly ten months prior to coming to us. Upon arrival at our clinic, bronchoscopic findings showed the left main bronchial stump was filled with blood and the mucous membrane of it was rough (Figure 1). The pathologic result indicated adenosquamous cell carcinoma on the basis of forceps biopsy by means of bronchoscopy. Cephalosome, chest and abdomen computed tomography showed nothing noteworthy (Figure 2). He was initially to be referred to receiving expectant treatment, but it was believed that he would not be able to tolerate a long-term therapy, because asphyxiation or sudden death could occur at any time in the course of treatment. Carinal resection and reconstruction with ECMO assisted venting was planned finally. While the preparation for ECMO was going on, an ultrasound examination was made to measure the internal diameter of the right common femoral artery and ruled out any vascular abnormalities.

The operation started with the placement of the arterial and venous cannulas under local anesthesia, first into the femoral artery and then into the homolateral vein. The cannulas had been flushed with a bolus of about 60 mL of heparinized saline solution (heparin doses 15 IU/kg body weight), and clamped proximally. The cannulas were then connected to the mechanical ventilation (Medtronic Bio-console 560) which was filled with sodium chloride physiological solution. When all the interface were well secured, the device was unclamped gently and progressively for 2 minutes to minimize the hemodynamic effects. After that, oxygen source was attached and the labeled oxygen inflow port with a line, raise the flow rate from 1 L/min to 8 L/min gradually, blood flow rate through the device fluctuated at 2.8 L/min to 3.1 L/min.

The posterolateral thoracotomy was carried out followed by intravenous anesthesia, which was induced with remifentanil, propofol, Midazolam, vecuronium and was maintained with target-controlled infusion of propofol (4 mg/kg.h) and remifentanil (1.5 mcg/kg plus 0.5-1.0 mcg/kg bolus at hourly intervals). The mediastinal pleura at the hilum was then completely incised. The inferior pulmonary ligament was incised up/down till to the inferior pulmonary vein, and thus, the lung was totally mobilized for the next step to anastomose stump.

Liberation and dissection of the right bronchus and trachea respectively proximal to the eminence about 2 cm were done first. The tracheal bronchiola anastomosis was done next. The membranous portion was attached with a 3-0 polydioxanone surgical (PDS) running suture, and the cartilaginous portion of the bronchus was closed with interrupted 3-0 PDS sutures that were spaced to accommodate the size difference between the trachea and primary bronchus. At the completion of the inosculation, the suture line was tested under water to preclude any air leaks, with the help of the anesthesiologist. Hereafter, the eminence and tracheal bronchiolar stumps were removed completely.

A single-lumen tube was inserted into the right main bronchus under endoscopic view via oral cavity and it was connected to the breathing machine. Ecmo was discontinue 1 hour postoperative when the vital sign of the patient was normal (BP: 139/86 mmHg, HR: 81/min, SPO_2_:94%, PaO_2_:92 mmHg, PaCO_2_:42 mmHg). Soon after that, the patient was transferred to the intensive care unit for respiratory care and early mobilization. He was discharged from the intensive care unit on postoperative day five and from the hospital on postoperative day ten.

## Discussion

Lung cancer recur and metachoresis to the bronchial stump is a life-threatening circumstance after pneumonectomy, carrying high mortality, and conventional treatment strategies for it were thought to provoke a high risk, because the general condition of the patient is usually poor, his chemosensitivity or radiosensitivity is low, and the contralateral main bronchus is the only gallery for life. To find an ideal therapeutic model is the pursuit of all chirurgeons all the time. Luckily, for the patient in question, we successfully applied ECMO, which is routinely used in cardiac surgery [1], to assist the carinal resection and reconstruction after left pneumonectomy.

As it is known to all, to maintain normal gas exchange intraoperatively is a real challenge to the performance of carinal resection and reconstruction, after pneumonectomy in particular. Conventional intubation to the contralateral respiratory tract is impossible while homolateral respiratory passage is absent, which makes oxygenation impossible. There are reports of options for airway management during operation, including apnea with intermittent bag mask ventilation, the use of a ventilating bronchoscope, jet, or spontaneous ventilation with suspension laryngoscopy, and the use of a laser-protected endotracheal tube with ventilation [2]. However, it is dangerous to adopt any one of the above options which are extremely challenging in the presence of active bleeding in the bronchus stump. In this case, as presented above, our goal was to keep regular gas exchange while with surgical field unobstructed during operation. ECMO is the safest option in managing the airway and maintaining gas exchange. By removing blood from the patient and circulating it through an artificial lung with a pump, ECMO can provide pulmonary and cardiac support, depending on the cannula arrangement [3], which can help achieve gas exchange despite the collapsed right lung and thus provide a clear unobstructed surgical field.

Some technical skills are required for this procedure. First, the ECMO blood flow rate should be adjusted so as to retain the patient's arterial oxygen saturation (SaO2) at 85-95% [4], because if ECMO circuit flow was too low, there would be significant blood flow through the nonventilated lungs, which could cause upper body (coronary and cerebral) hypoxemia [5]. Second, the anaesthetic tube should be inserted to the right main bronchus after the anastomotic stoma was done well, which would save the operative time with ECMO assistance. On the contrary, if the tube was inserted before or in the course of anastomosis, it would prolong the time exposure to ECMO and hinder the operation procedure. Third, the anaesthetic tube must be inserted to the right main bronchus under endoscopic and be connected to the respirator, which would avoid the damage to the vulnerable stoma and assure the patient's safety when the ECMO is disconnected.

Based on the successful outcome, we consider that ECMO might become a new reliable appliance for tracheasurgery, especially in carinal resection and reconstruction after pneumonectomy.

## Abbreviations

ECMO: Extracorporeal membrane oxygenation

## Competing interests

The authors declare that they have no competing interests.

## Authors' contributions

JL was a master in the thoracic surgery in Tang DU hospital, and wrote most part of this manuscript. XPW was an associate professor in Tang DU hospital, also was a chief operator in the operation, and gave some comments for this manuscript. KS, YA Z, YH, LJH were assistant operators in the operation. XFL was a general supervisor of this manuscript.

All the authors read and approved the final manuscript.

## Consent

Written informed consent was obtained from the patient for publication of this case report and accompanying images. A copy of the written consent is available for review by the Editor-in-Chief of this journal.

## References

[B1] Marasco SF Lukas G McDonald M Review of ECMO (Extra Corporeal Membrane Oxygenation) Support in Critically Ill Adult Patients Heart, Lung and Circulation 2008 17 41 47 10.1016/j.hlc.2008.08.009 18964254

[B2] Cohen E Neustein S Eisenkraft J Barash PG, Cullen BF, Stoelting RK Anesthesia for thoracic surgery, Clinical Anesthesia 2005 5 Philadelphia, Lippincott Williams Wilkins 838 9

[B3] Schuerer DJ Kolovos NS Boyd KV Extracorporeal Membrane Oxygenation Chest 2008 134 179 184 10.1378/chest.07-2512 18628221

[B4] Smith IJ Sidebotham DA McGeorge AD Use of Extracorporeal Membrane Oxygenation during Resection of Tracheal Papillomatosis Anesthesiology 2009 110 427 9 19194169 10.1097/ALN.0b013e3181943288

[B5] Beca J Wilcox T Hall R Sidebotham D, McKee A, Gillham M, Levy J Mechanical Cardiac Support, Cardiothoracic Critical Care Philadelphia Butterworth Heinmann Elsevier 2007 1 672

